# High-intensity focused ultrasound (HIFU) and low-intensity focused ultrasound (LIFU) as neurosurgical approaches in psychiatry: A systematic review of current clinical evidence

**DOI:** 10.1016/j.bas.2026.107438

**Published:** 2026-09-07

**Authors:** Alberto Cervantes-Hernández, Ailyn Montúfar Medrano, Humberto Nicolini, Fabián César Piedimonte, Rosalia Zerón-Martínez, Fiacro Jimenez-Ponce

**Affiliations:** a Facultad Mexicana de Medicina, Universidad La Salle, Mexico City, Mexico; b Centro de Neurociencias, Hospital Angeles Pedregal, Mexico City, Mexico; c Instituto Nacional de Medicina Genómica, Mexico City, Mexico; d Hospital Central Militar, Mexico City, Mexico; e Fundación CENIT, University of Buenos Aires, Argentina

**Keywords:** High-intensity focused ultrasound, Low-intensity focused ultrasound, Neuropsychiatric disorders, Neuromodulation, Transcranial focused ultrasound

## Abstract

**Objectives:**

Transcranial focused ultrasound (tFUS), including high-intensity focused ultrasound (HIFU) and low-intensity focused ultrasound (LIFU), has emerged as an incisionless approach for modulating or ablating brain circuits implicated in psychiatric disorders. This systematic review summarizes the current clinical evidence on HIFU and LIFU for psychiatric indications, with emphasis on clinical outcomes, safety, targets, and methodological limitations. However, HIFU has primarily been investigated for incisionless ablative procedures, whereas LIFU has mainly been explored as a reversible neuromodulation technique. Given these distinct therapeutic objectives, direct comparison between both modalities should be interpreted cautiously.

**Materials and methods:**

A systematic literature search was conducted in PubMed/MEDLINE, Embase, Scopus, and Cochrane for studies published between 2014 and 2025 evaluating HIFU and LIFU in adults with psychiatric or neuropsychiatric disorders. Clinical trials, pilot studies, open-label studies, and sham-controlled studies reporting clinical outcomes, stimulation or ablation targets, and adverse events were included. Studies involving nonpsychiatric indications, animal models, reviews, editorials, protocols, or other neuromodulation techniques that did not use focused ultrasound as the primary intervention were excluded.

**Results:**

Eight studies met the inclusion criteria, comprising three HIFU and five LIFU studies. HIFU studies primarily evaluated magnetic resonance-guided bilateral capsulotomy targeting the anterior limb of the internal capsule in treatment-refractory obsessive-compulsive disorder and major depressive disorder. LIFU studies evaluated nonablative neuromodulation of fronto-limbic targets in mood, anxiety, trauma-related, and psychotic disorders. Across studies, focused ultrasound was associated with improvements in several psychiatric rating scales. Reported adverse effects were generally mild and transient, most commonly headache, dizziness, scalp sensitivity, or procedure-related discomfort. However, evidence was limited by small sample sizes, heterogeneous diagnoses, variable follow-up, and risk of bias.

**Conclusions:**

Preliminary clinical evidence suggests that HIFU and LIFU may be associated with symptom improvement in selected psychiatric populations. Larger randomized, sham-controlled, multicenter trials are required to define efficacy, safety, optimal targets, and clinical implementation.

## Highlights

• Focused ultrasound showed symptom improvement in psychiatric disorders.
• High-intensity focused ultrasound (HIFU) was useful for targeting severe obsessive-compulsive disorder and treatment-resistant major depressive disorder.
• Low-intensity focused ultrasound (LIFU) modulated mood and anxiety brain circuits.
• Most adverse effects were mild, transient, and procedure-related.
• MRI-guided bilateral thermal capsulotomy was useful for targeting the anterior limb of the internal capsule (ALIC).

## 1 Introduction

**Fig. 1:**
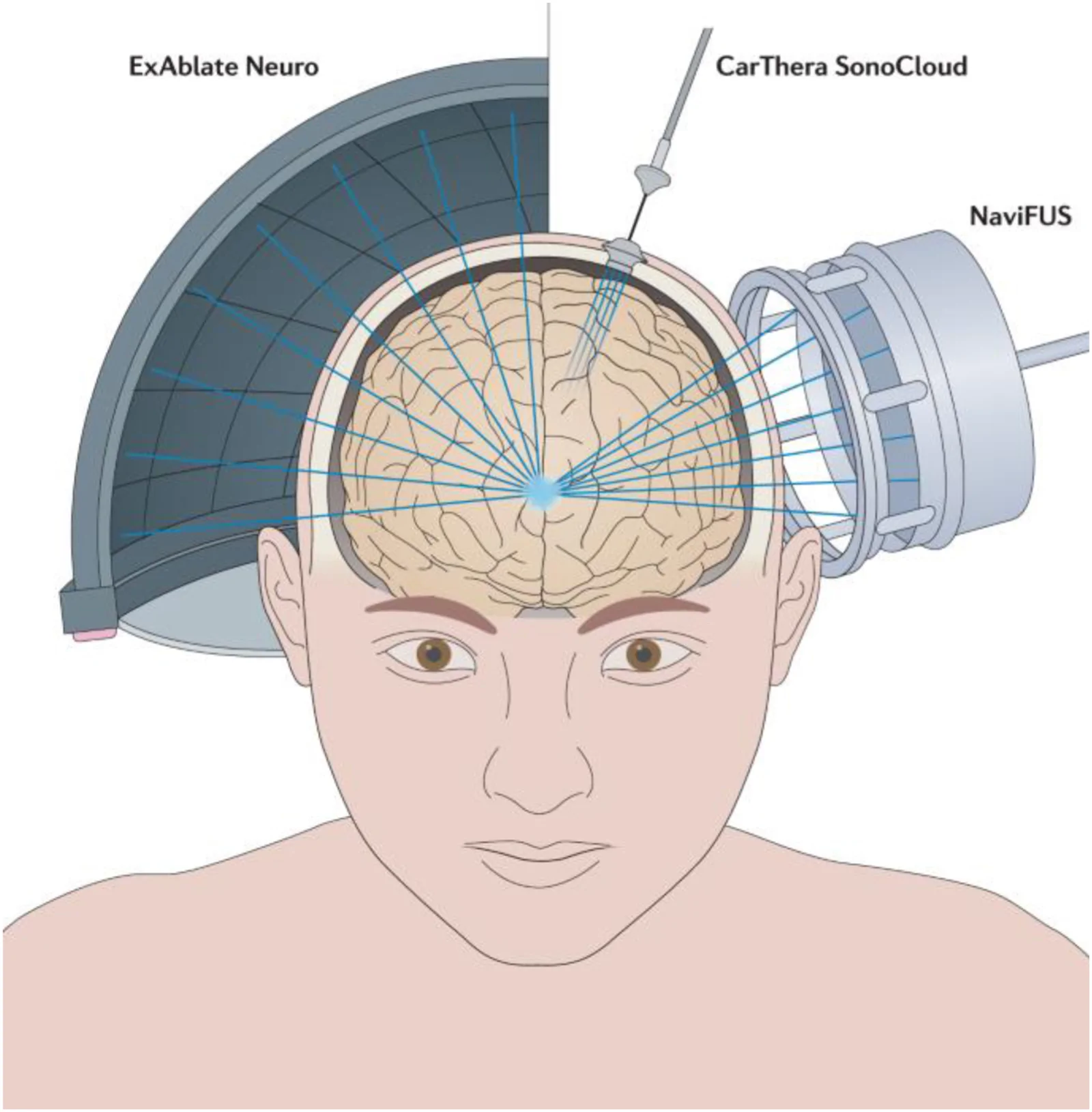
Schematic representation of transcranial focused ultrasound systems used for neuromodulation. The ExAblate Neuro (left) delivers **MR-guided high-intensity focused ultrasound (HIFU)** through a hemispheric transducer array for precise intracranial targeting. The CarThera SonoCloud (center) and NaviFUS (right) represent alternative ultrasound platforms. Focused acoustic beams converge at a single focal point, producing controlled thermal and mechanical effects for therapeutic purposes (Meng et al., 2021).

In the past, treatments for neuropsychiatric disorders like major depressive disorder, anxiety disorders, and chronic pain were limited to psychopharmacological and psychotherapeutic approaches; however, recent technological advances have introduced treatments targeting brain neuroanatomical circuits, leading to the development of new neuromodulation techniques, which, over the past 25 years, have successfully modified neural substrate activity (Cox et al., 2025). High-intensity focused ultrasound (HIFU) is a novel incisionless technique in neurosurgery and neuromodulation with potential for treating neuropsychiatric disorders. It precisely targets deep brain regions involved in psychiatric disorders, producing permanent changes via thermoablation or temporary effects via neuromodulation, depending on the ultrasound intensity (Shah et al., 2025; Davidson et al., 2025). The other of these therapies is Low-Intensity Focused Ultrasound (LIFU), which differs from HIFU due to its reversible neuromodulatory capacity at lower intensities (Arulpragasam et al., 2022). LIFU is highly controllable, precise, and incisionless therapeutic option when treating psychiatric disorders (Badran and Peng, 2024; di Biase et al., 2019). Although both modalities rely on focused ultrasound, HIFU and LIFU differ substantially in their mechanisms of action and therapeutic goals. Therefore, this review summarizes the current evidence for each technique separately rather than establishing direct superiority between them (see Table 4, Fig. 1).

Despite the existence of neuromodulation techniques like deep brain stimulation (DBS) and vagus nerve stimulation (VNS) for multi-drug-resistant patients, there exist possible complications like infection, intracranial hemorrhage, misplacement of hardware, mood and cognitive changes, dysarthria, paresthesias, gait/balance issues, nerve dysfunction, or cardiopulmonary affections (Kähkölä et al., 2024).

Given the previously mentioned advantages of transcranial focused ultrasound (tFUS = HIFU or LIFU) technology, and the substantial demand for mental disorder treatments, many researchers have been actively investigating its application and mechanisms in psychiatric conditions such as depression, anxiety, schizophrenia, and substance use disorders (SUDs) over the past five to ten years (Shi and Wu, 2025); however, it is not yet fully comprehended its action mechanism in the brain's cytochemistry, nor it is officially approved as a unique treatment. It should be taken with reserve until there are more studies about the subject.

HIFU concentrates multiple ultrasound waves into a single focal point within the tissue, producing biological effects through thermal and mechanical processes, of which the latter is not yet fully understood. In the applied tissue, acoustic energy converts to heat, rapidly raising local temperatures from 55 °C to 65 °C, inducing coagulative necrosis and protein denaturation while sparing surrounding tissues due to the focal energy gradient (Quadri et al., 2018). This thermal mechanism causes tissue ablation (thermoablative effect) in procedures like thalamotomy or tumor destruction. HIFU's mechanical effects, including acoustic cavitation, radiation force, and microstreaming, generate shear stresses and bubble dynamics that disrupt cell membranes and microvasculature, causing tissue damage and neuromodulatory effects at subablative levels (Kähkölä et al., 2024).

LIFU performs with a temperature rise of only <1–2 °C above the standard human temperature. The main effect is hypothesized to be mechanical modulation of neuronal membranes, ion channels, and synaptic transmission, leading to a reversible effect rather than ablation, though the specific action mechanism has not yet been discovered. Unlike HIFU, which is primarily designed to produce thermal lesions or, in selected applications, transiently open the blood–brain barrier to facilitate targeted drug or gene delivery, LIFU is being investigated as a non-ablative neuromodulation technique. Because HIFU and LIFU differ fundamentally in their mechanisms of action and intended therapeutic objectives, they should be regarded as complementary focused ultrasound modalities rather than directly comparable interventions. Ongoing technological advances, including MRI guidance, phased-array transducers, and real-time acoustic monitoring, continue to improve the precision and safety of focused ultrasound applications (Kähkölä et al., 2024; Jung et al., 2015a).

Several articles have explored the application of HIFU and LIFU. We included 8 articles published between 2014 and 2025. The most common psychiatric condition studied was OCD, followed by depression, substance use disorders (SUDs), and anxiety.

This systematic review aims to provide a descriptive overview of an early-stage field on transcranial focused ultrasound in psychiatry, describing high-intensity ablative approaches and low-intensity neuromodulatory approaches. Specifically, we summarize psychiatric indications, neural targets, clinical outcomes, adverse effects, and risk of bias across available human studies. Given the early stage of the field, this review is intended to clarify current evidence gaps and inform future clinical trial design rather than establish definitive therapeutic recommendations.

## 2 Materials and Methods

This systematic review was conducted according to the Preferred Reporting Items for Systematic Reviews and Meta-Analyses (PRISMA) 2020 statement. The review protocol was registered in PROSPERO under registration number CRD420261307004.

A systematic literature search was conducted in PubMed/MEDLINE, Embase, Scopus, and Cochrane for studies published from January 1, 2014, to December 31, 2025. The search strategy combined terms related to focused ultrasound and psychiatric disorders. Search terms included combinations of: “focused ultrasound,” “transcranial focused ultrasound,” “high-intensity focused ultrasound,” “low-intensity focused ultrasound,” “HIFU,” “LIFU,” “MR-guided focused ultrasound,” “MRgFUS,” “transcranial ultrasound stimulation,” “psychiatric disorder,” “neuropsychiatric disorder,” “obsessive-compulsive disorder,” “major depressive disorder,” “depression,” “anxiety,” “generalized anxiety disorder,” “schizophrenia,” “substance use disorder,” and “trauma-related disorder.” Reference lists of included articles and relevant reviews were manually screened to identify additional eligible studies.

Inclusion criteria.

● Human participants diagnosed with neuropsychiatric or psychiatric disorders like obsessive-compulsive disorder (OCD), treatment-refractory OCD, major depressive disorder (MDD), treatment-resistant generalized anxiety disorder, schizophrenia's negative symptoms, mood, anxiety, and trauma-related disorders (MATRDs).
● The population characteristics were patients aged 18 years and older
● Studies evaluating HIFU or LIFU as a therapeutic or investigational intervention targeting specific brain areas.
● Studies reporting clinical, behavioral, neurophysiological, or imaging outcomes related to psychiatric symptom improvement, neural modulation, safety, or feasibility.
● Study design: clinical trials, open-label design, double-blind pilot study, sham-controlled clinical trial.
● Studies published between 2014 and 2025
● Clinimetric scores for evaluation of the disease
● Adverse events reported

Exclusion criteria.

● Studies involving non-psychiatric indications
● HIFU and LIFU were administered on animals
● Other neuromodulation techniques without HIFU as the primary intervention (e.g., DBS, rTMS, tDCS, VNS).
● Publication types: Reviews, editorials, commentaries, or conference abstracts without primary data.
● Protocols or theoretical papers lacking experimental or clinical evidence. Duplicate publications of the same dataset.

Studies were grouped by transcranial focused ultrasound modality, distinguishing between HIFU and LIFU. Within each group, outcomes related to clinical efficacy were included. This was measured using psychiatric scales, administered before and months after the procedure, with varying time gaps across studies. The most common adverse events were included from each trial.

Titles and abstracts were screened independently by two reviewers using Rayyan-AI (Ouzzani et al., 2016). Full-text articles were then assessed for eligibility. Disagreements were resolved by consensus with a third reviewer. Extracted data included author, year, study design, sample size, diagnosis, focused ultrasound modality, neural target, stimulation or ablation parameters when available, clinical outcome measures, follow-up duration, main clinical outcomes, and reported adverse events.

Given the heterogeneity of diagnoses, ultrasound modalities, targets, outcome scales, and follow-up periods, a quantitative meta-analysis was not performed. Findings were synthesized narratively and grouped by focused ultrasound modality: HIFU/MRgFUS (MR-guided Focused Ultrasound) and LIFU/transcranial ultrasound stimulation.

## 3 Risk of bias assessment

The clinical trials included in this review were evaluated using the Cochrane Risk of Bias tool version 2 (RoB-2). These were the domains assessed: bias arising from the randomization process, bias due to deviations from intended interventions, bias due to missing outcome data, bias in measurement of the outcome, and bias in selection of the reported result. Each domain was categorized as Low risk, Some concerns, or High risk of bias, according to RoB-2 guidance. Also, an overall risk of bias judgment was assigned for each study. Non-randomized HIFU studies were not eligible for RoB-2 assessment due to the absence of randomization and control groups. Therefore, these studies were considered to have an inherently high risk of bias due to their study design and instead were assessed for quality control by The National Institutes of Health (NIH) Quality Assessment Tool for Before–After (Pre–Post) Studies With No Control Group. Each study was assessed and evaluated with the previously mentioned tools by three investigators, and after that, a consensus was reached to agree on the same categories applied to all studies.

**Fig. 2:**
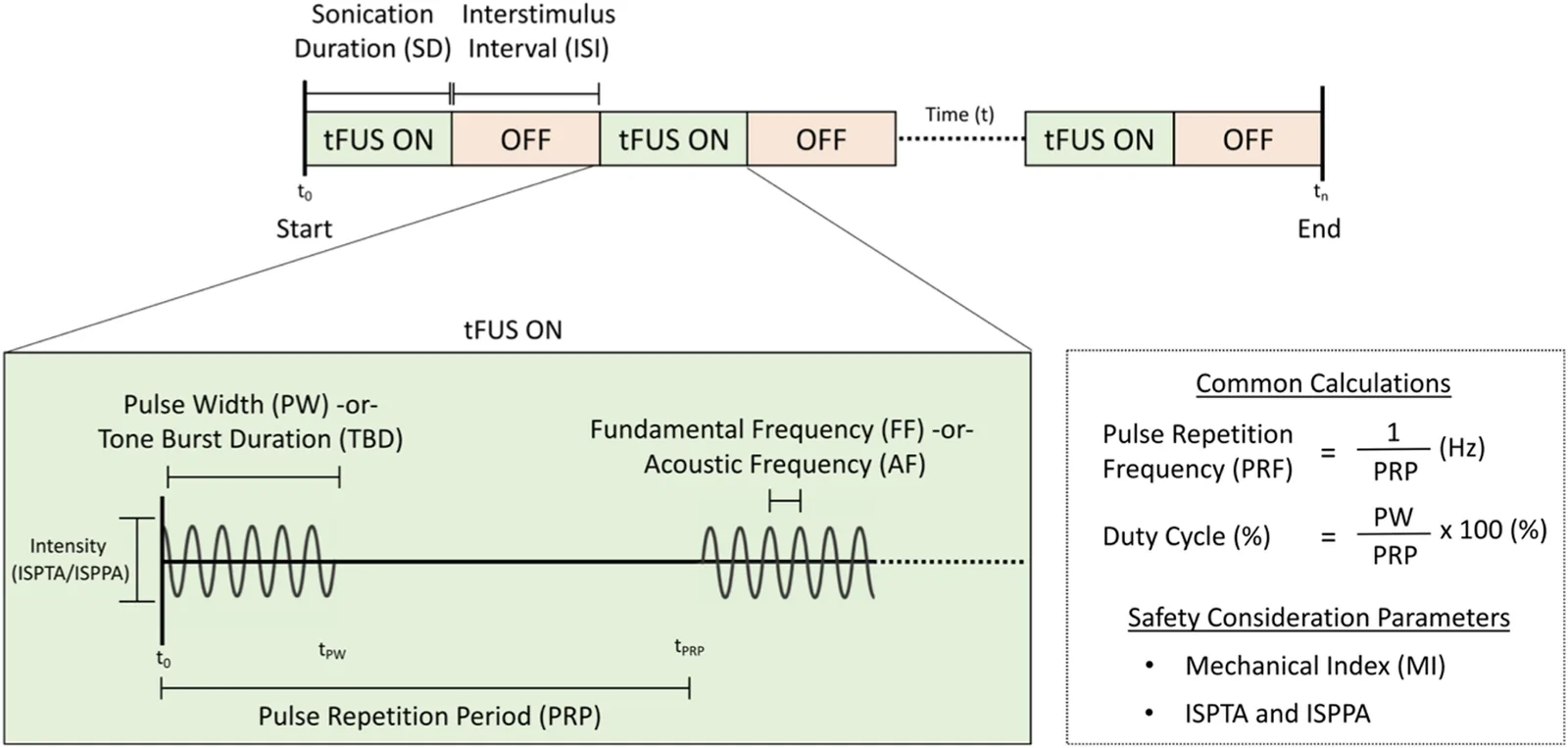
Schematic of primary transcranial focused ultrasound (tFUS) stimulation parameters. This diagram illustrates key pulsing variables used during tFUS, including sonication duration (SD), interstimulus interval (ISI), pulse width (PW) or tone burst duration (TBD), pulse repetition period (PRP), and fundamental/acoustic frequency (FF/AF). Commonly derived parameters—pulse repetition frequency (PRF) and duty cycle (%)—are also shown, along with safety indices such as Mechanical Index (MI) and intensity measures (ISPTA, ISPPA). These parameters collectively determine the thermal and mechanical bioeffects of ultrasound neuromodulation (Cox et al., 2025).

This study was carried out during the previously mentioned period. The records identified from databases and registers are the following: PubMed/MEDLINE: 14, Embase: 9, Scopus: 8, Cochrane Library: 2, Clinical trial registers/manual searching: 5. This makes a total number of records identified of 38 (see Fig. 2).

**Fig. 3:**
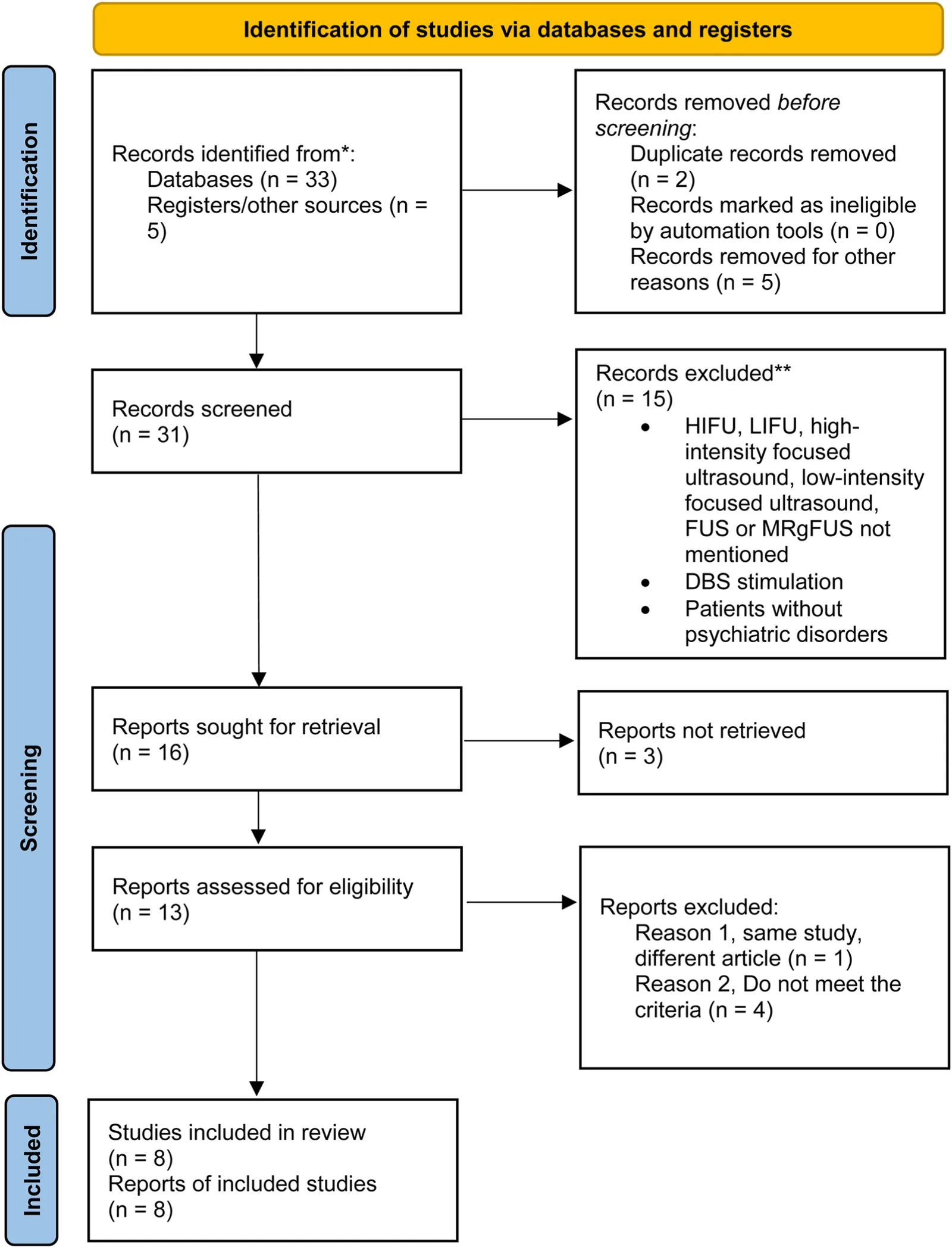
PRISMA flowchart for the identification and screening of HIFU and LIFU studies for systematic search. Data added to the PRISMA template (from Page MJ, McKenzie JE, Bossuyt PM, Boutron I, Hoffmann TC, Mulrow CD et al. The PRISMA, 2020 statement: an updated guideline for reporting systematic reviews. BMJ. 2021; 372:n71) under the terms of the Creative Commons Attribution (CC BY 4.0) License (https://creativecommons.org/licenses/by/4.0/).

In Fig. 3, the PRISMA diagram describes the algorithm of the papers.

## 4 Results

All the study characteristics were synthesized in a table format, so the information can be displayed visually and compared between them. A set of 9 groups was created to set the characteristics sought by each of the studies included. Among the created characteristic groups, only two studies were missing the LIFU parameters, which were omitted in the synthesis. Table 1 shows the results of the clinical effects of HIFU in psychiatric disorders, and 3 studies were found eligible. In these studies, OCD was the main disease studied.

**Table 1:** Clinical characteristics and outcomes of HIFU/MRgFUS studies in psychiatric disorders. This table consists of three clinical trials, in which HIFU was applied to patients with OCD and MDD. The brain structure ALIC was targeted, and several psychiatric scales were applied before and after the procedure. Adverse effects were also included.

Article Name	Principal Author	Year	Type of study	n (patients)	Participants Diagnosis	Target and parameters	Scales	Clinical Outcomes Scores (before and after HIFU)	Adverse Effects
Bilateral thermal capsulotomy with MR-guided focused ultrasound for patients with treatment-refractory obsessive-compulsive disorder: a proof-of-concept study (Arulpragasam et al., 2022)	Jung HH	2014	Clinical trial	4 patients	OCD◻	**ALIC:** 7 mm anterior to the anterior margin of the anterior commissure at the same AC–PC plane, extending 2–3 mm along the capsule from a coronal view. MRgFUS was performed in a 3-T MR imaging (MRI) system (GE Medical System, Milwaukee, WI, USA) using the ExAblate 4000 (InSightec, Haifa, Israel)	Y-BOCS HAM-D HAM-A	**Y-BOCS:** baseline: 35.3 ± 1.9 at 6 months: 23.5 ± 4.9 = Δ −11.8 **HAM-D:** baseline: 22.5 ± 4.2 at 6 months: 8.8 ± 3.3 = Δ −13.7 **HAM-A:** baseline: 27 ± 7.4 at 6 months 8.3 ± 6.0 = Δ −18.7	• Mild short headaches • Vestibular symptoms (nausea, vomiting or dizziness) • Weight gain
						Parameters: Temperature in the target region of 51–56 °C, applied for more than 3 s. A 10-mm elliptical lesion was created by the target center. Number of sonications ranged from 23 to 36 hz, with 10–31 s durations. Duration of the procedure: 5–7 h			
A study of novel bilateral thermal capsulotomy with focused ultrasound for treatment-refractory obsessive– compulsive disorder: 2-year follow _up_ 23	Kim SJ	2018	Open-label design	11 patients	Treatment-refractory OCD	**ALIC:** 7 mm in front of the anterior margin of the anterior commissure. MRgFUS was performed in a 3-T MR imaging (MRI) system (GE Medical System, Milwaukee, WI, USA) using the ExAblate 4000 (InSightec, Haifa, Israel). Parameters: Temperature of 51 °C to 56 °C for more than 3 s. A10 mm elliptical lesion along the internal capsule was created.	Y-BOCS HAM-D HAM-A GAF CGI-S CGI-I	**Y-BOCS:** baseline-34.4 ± 2.3 at 24 months-21.3 ± 6.2 = Δ −13.1 **HAM-D:** baseline-19.0 ± 5.3 at 24 months-7.6 ± 5.3 = Δ −11.4 **HAM-A:** baseline-22.4 ± 5.9 at 24 months-7.9 ± 3.9 = Δ −14.5 **GAF:** baseline-35.8 ± 4.9 at 24 months-56.0 ± 10.3 = Δ 20.2 **CGI-S:** baseline-6.1 ± 0.2 at 24 months-3.9 ± 0.3 = Δ −2.2 **CGI-I:** baseline-3.4 ± 0.2 at 24 months-2.1 ± 0.3 = Δ −1.3	• **Headache:** The most common adverse event, experienced by seven patients (63.6%), was short, periodic headaches during sonication at temperatures > 50 °C. **Vestibular Symptoms:** Five patients (45.5%) had increased vestibular symptoms, such as nausea, vomiting, or dizziness, which continued until the end of the procedure. • **Anxiety:** Three patients (27.3%) felt increased anxiety. • **Stomach Upset:** Two patients (18.2%) complained of stomach upset. • **Warm Sensation:** One patient (9.1%) experienced a transient warm sensation in the brain during sonication at higher temperatures.
Magnetic resonance-guided focused ultrasound capsulotomy for refractory obsessive compulsive disorder and major depressive disorder: clinical and imaging results from two phase I trials (Germann et al., 2021)	Davidson B	2020	Open-label series	OCD: 6 patients MDD: 6 patients	OCD and MDD	**ALIC:** ∼7 mm anterior to the anterior commissure's posterior border, and 12 mm lateral from the midline. MRgFUS was performed in a 3-T MR imaging (MRI) system (GE Medical System, Milwaukee, WI, USA) using the ExAblate 4000 (InSightec, Haifa, Israel)	HAM-D YBOCS (not measured in MDD patients)	**YBOCS** among all OCD patients: baseline: 33.0 (±7.6) at 6 months: 22.0 (±8.9) = Δ −11 **HAMD17:** baseline 24.67 (±2.42) at 6 months 17.83 (±11.05) = Δ −6.84	• Erythema at pin sites • Mild headaches • Supraorbital pin-site swelling • Scalp sensitivity
						Parameters: Temperature in the target region over 53 °C Duration of the procedure: 3–4 h			
Resume	NA	NA	NA	27 patients	OCD/MDD	**ALIC**	Y-BOCS HAM-D HAM-A	**Y-BOCS:** Average −11.96 **HAM-D:** Average −10.64 **HAM-A:** Average −16.6 **GAF:** 20.2 **CGI-S:** −2.2 **CGI-I:** −1.3	The two most common adverse effects in all the studies were headaches and vestibular symptoms.

OCD = Obsessive-Compulsive Disorder; MDD = Major Depressive Disorder (MDD); ALIC = Bilateral Anterior Limb of the Internal Capsule; Y-BOCS = Yale-Brown Obsessive-Compulsive Scale; HAM-D = Hamilton Rating Scales for Depression; HAM-A = Hamilton Rating Scales for Anxiety; GAF = Global Assessment of Functioning; CGI-S = Clinical Global Impression – Severity; CGI-I = Clinical Global Impression-Improvement; NA = Not Applicable.

Table 1 summarizes the clinical results of these trials on the use of HIFU as a neurosurgical intervention for treatment-refractory psychiatric disorders, primarily OCD and MDD. It includes three clinical studies published between 2014 and 2020, encompassing 27 patients who underwent MRI-guided bilateral thermal capsulotomy targeting the ALIC. Study designs consisted of small clinical trials and open-label series, reflecting the exploratory nature of this therapeutic approach. Clinical outcomes were assessed using standardized psychiatric rating scales, including the Yale–Brown Obsessive Compulsive Scale (Y-BOCS), Hamilton Depression Rating Scale (HAM-D), Hamilton Anxiety Rating Scale (HAM-A), Global Assessment of Functioning (GAF), and Clinical Global Impression (CGI) scales.

In Jung et al., significant reductions were observed across all clinical measures at 6 months, including Y-BOCS (Δ −11.8), HAM-D (Δ −13.7), and HAM-A (Δ −18.7), indicating substantial improvements in obsessive-compulsive, depressive, and anxiety symptoms. The second study of Kim et al. had sustained improvements at 24 months across all outcome measures, including reductions in Y-BOCS (Δ −13.1), HAM-D (Δ −11.4), and HAM-A (Δ −14.5), accompanied by significant gains in global functioning (GAF Δ +20.2) and clinician-rated severity and improvement (CGI-S Δ −2.2; CGI-I Δ −1.3). In the final study by Davidson et al., which analyzed both OCD and MDD, Y-BOCS scores decreased from 33.0 ± 7.6 at baseline to 22.0 ± 8.9 at 6 months (Δ −11), indicating a substantial reduction in obsessive symptoms severity. HAM-D17 scores also decreased from 24.67 ± 2.42 to 17.83 ± 11.05 (Δ −6.84), reflecting moderate improvement in depressive symptoms. Reported adverse effects were generally mild and transient, with the most common being headache and vestibular symptoms, and no severe or permanent neurological complications were described.

Table 2summarizes the current clinical evidence on the use of low-intensity focused ultrasound (LIFU) as an incisionless neuromodulation strategy for psychiatric disorders, compiling data from five studies published between 2020 and 2025, and including 138 participants. The studies comprise predominantly double-blind randomized controlled trials, along with open-label pilot designs, and evaluate LIFU across a heterogeneous set of diagnoses, including mild to moderate depression, major depressive disorder, treatment-resistant generalized anxiety disorder, schizophrenia with predominant negative symptoms, and mood, anxiety, and trauma-related disorders (MATRDs). Neuromodulation targets vary according to the clinical condition. The areas targeted by the researchers were the right fronto-temporal cortex, left dorsolateral prefrontal cortex (DLPFC), and bilateral or unilateral amygdala, reflecting the involvement of fronto-limbic circuits in affective and cognitive regulation (see Table 3).

**Table 2:** Clinical characteristics and outcomes of LIFU/transcranial ultrasound stimulation studies in psychiatric disorders. The following table summarizes the clinical outcomes scores of participants with psychiatric conditions when treated with LIFU, as well as their adverse events.

Articles	Author	Year	Type of study	n (patients)	Participants Diagnosis	Target and parameters	Scales	Clinical Outcomes Scores (before and after HIFU)	Adverse events
A double-blind pilot study of transcranial ultrasound (TUS) as a five-day intervention: TUS mitigates worry among depressed participants (Mahdavi et al., 2023)	Reznik SJ	2020	Double-blind pilot study	12 patients	Mild to moderate depression	**RFTC:** specifically using electrode site F8. TUS neuromodulation was performed using a Neurotrek U+™, an ultrasound device developed by Neurotrek Inc., Los Gatos, CA. Parameters: Power 11% Frequency of 0.5 MHz, Duration of exposure of 30 s. The speed of sound entered was 1550 m/s. Brain density was 1030 kg/m^3. There was a 53% reduction in field intensity through the skull.	BDI-II OASIS RRS PSWQ SCID-5	**BDI-II:** average baseline 17.33 ± 1.214 at 1 month F(1, 12) = 0.196, p = 0.667, ηp 2 = 0.019 **OASIS:** average baseline 5.75 ± 0.986 at 1 month F(1, 12) = 4.422, p = 0.062, ηp 2 = 0.307 **PSWQ:** average 57.75 ± 2.758, no results reported at 1 month **RRS:** average 49.92 ± 2.530, no results reported at 1 month	None
A pilot study of low-intensity focused ultrasound for treatment-resistant generalized anxiety disorder (Zhai et al., 2023)	Mahdavi KD	2023	Open-label pilot study	25 patients	Treatment-resistant GAD	**RA:** TUS neuromodulation was performed using a Brainsonix Pulsar 1002 device. Parameters: Number of applications: 8 consecutive weekly Frequency at 650 kHz Pulse width of 5 ms Duty cycle of 5% Pulse repetition frequency of 10 Hz ISPPA.3 of 14.39 W/cm2 and an ISPTA.3 of 719.73 mW/cm2 Duration 30 s on and 30 s off for 10 min of 10-min focused ultrasound sessions.	HAM-A BAI PGI-I	**HAM-A:** baseline 33.16 at 2 months 20.52 = Δ - 12.64 **BAI:** baseline 28.84 at 2 months 15.96 = Δ - 12.88 **PGI-I:** at 2 months 2.16	None
The efficacy of low-intensity transcranial ultrasound stimulation on negative symptoms in schizophrenia: A double-blind, randomized sham-controlled study (Martínez-Fernández et al., 2020)	Zhai Z	2023	Double-blind randomized sham-controlled study	26 patients	SNS	**DLPFC:** TUS neuromodulation was performed using a transducer (V391-SU, Olympus NDT, Waltham, USA)	PANSS SANS C-BCT	**SANS:** t = 7.397, P < 0.001 **PANSS:** F = 4.503, P < 0.05, Partial n^2^ = 0.158 **C-BCT:** F = 4.277, P = 0.050	• **Difficulty falling asleep:** 2 of 13 patients in the active group. This symptom resolved within 7 days. • **Dizziness:** Felt by 3 cases in the sham group throughout the intervention
Effect of Low-Intensity Transcranial Focused Ultrasound Stimulation in Patients With Major Depressive Disorder: A Randomized, Double-Blind, Sham- Controlled Clinical Trial (Barksdale et al., 2025)	Oh	024	Randomized, Double- Blind, Sham-Controlled Clinical Trial	23 patients	MDD	**Left DLPFC**	MADRS QIDS-SR SSI K-POMS	**MADRS:** baseline 28.5 ± 8.4 - at 2 weeks 14.8 ± 7.2 = Δ −13.7 **QIDS-SR:** baseline 21.7 ± 6.5 - at 2 weeks 10.8 ± 7.0 = Δ −10.9 **SSI:** baseline 15.1 ± 9.6 - at 2 weeks 5.5 ± 6.5 = Δ – 10 **K-POMS:** baseline 115.5 ± 34.6 - at 2 weeks 54.7 ± 35.5 = Δ −60.8	None
Low-intensity transcranial focused ultrasound amygdala neuromodulation: a double-blind sham controlled target engagement study and unblinded single-arm clinical trial (Risk of bias tools)	Barksdale BR	2025	Double-blind clinical trial	29 patients with MATRDs and 23 healthy comparison subjects	MATRDs	**LA**	MASQ-GD PHQ-9 GAD-7 SHAPS	**MASQ-GD:** baseline 26.79 ± 6.41 - at 3 weeks+3-7 days 21.10 ± 7.02 = Δ −5.69 **PHQ-9:** baseline 11.00 ± 5.29 - at 3 weeks+3-7 days 6.55 ± 4.75 = Δ - 4.45 **GAD-7:** baseline 11.31 ± 4.91 - at 3 weeks+3-7 days 6.80 ± 5.35 = Δ - 4.51 **SHAPS:** baseline 3.00 ± 3.60 - at 3 weeks+3-7 days 1.14 ± 2.82	• Headache Irritability • Decreased concentration Tingling in limbs and hands • Dizziness
Resume	5	2020-2025	Open-label pilot study N = 1, double-blind N = 4	138 patients	Mild to moderate depression Treatment-resistant GAD SNS MDD MATRDs	**RFTC RA DLPFC** **Left DLPFC LA**	Across these studies, validated psychometric instruments showed clinically meaningful improvements over follow- up periods from two weeks to two months. In contrast, some scales showed non-significant changes or lacked follow-up data.	Moderate to large improvements were observed over short- to mid-term follow-up, with favorable global clinical improvement.	• Headaches • Dizziness

RFTC = Right Fronto-Temporal Cortex; BDI-II = Beck Depression Inventory-II; OASIS = Overall Anxiety Severity and Impairment Scale; RRS = Ruminative Responses Scale; PSWQ = Penn State Worry Questionnaire; SCID-5 = Structured Clinical Interview for the DSM-5; GAD = Generalized Anxiety Disorder; RA = Right Amygdala; SNS: Schizophrenia's Negative Symptoms; DLPFC = Left Dorsolateral Prefrontal Cortex; LA = Left Amygdala; BAI = Beck Anxiety Inventory; PGI-I = Patient Global Impression-Improvement; PANSS = Positive and Negative Syndrome Scale; SANS = Scale for the Assessment of Negative Symptoms; C-BCT = Chinese Brief Cognitive Test; MADRS = Montgomery-Åsberg Depression Rating Scale; QIDS-SR = Quick Inventory of Depressive Symptomatology-Self Report; SSI = Scale for Suicide Ideation; K-POMS = Korean edition of the Profile of Mood States; MASQ-GD = Mood and Anxiety Symptom Questionnaire General Distress; PHQ-9 = Patient Health Questionnaire-9; GAD-7 = Generalized Anxiety Disorder-7; SHAPS = Snaith-Hamilton Pleasure Scale; MATRDs = Mood, Anxiety, and Trauma-Related Disorders.

**Table 3:** Risk of bias assessment of randomized and sham-controlled LIFU studies using the Cochrane RoB 2 tool (Risk of bias tools).

Study	Randomization Process	Deviations from Intended Interventions	Missing Outcome Data	Measurement of Outcome	Selection of Reported Results	Overall Risk of Bias
Reznik et, al. 2020	Some concerns	Low risk	Some concerns	Low risk	Some concerns	Some concerns
Mahdavi et, al. 2023	Low risk	Low risk	Some concerns	Low risk	Some concerns	Some concerns
Zhai et, al. 2023	Low risk	Low risk	Low risk	Low risk	Some concerns	Some concerns
Oh et, al. 2024	Low risk	Low risk	Low risk	Low risk	Low risk	Low risk
Barksdale et, al. 2025	Low risk	Low risk	Some concerns	Low risk	Some concerns	Some concerns

**Table 4:** Quality assessment of included HIFU studies using the NIH Quality Assessment Tool for Before–After (Pre–Post) Studies With No Control Group (NHLBI, 2013).

Criteria	Jung et al. (2015)	Kim et al. (2018)	Davidson et al. (2020c)
1. Was the study question or objective clearly stated?	Yes	Yes	Yes
2. Were eligibility/selection criteria for the study population prespecified and clearly described?	Yes	Yes	Yes
3. Were the participants in the study representative of those who would be eligible for the test/service/intervention in the general or clinical population of interest?	Yes	Yes	Yes
4. Were all eligible participants that met the prespecified entry criteria enrolled?	CD	CD	CD
5. Was the sample size sufficiently large to provide confidence in the findings?	No	No	No
6. Was the test/service/intervention clearly described and delivered consistently across the study population?	Yes	Yes	Yes
7. Were the outcome measures prespecified, clearly defined, valid, reliable, and assessed consistently across all study participants?	Yes	Yes	Yes
8. Were the people assessing the outcomes blinded to the participants' exposures/interventions?	No	No	No
9. Was the loss to follow-up after baseline 20% or less? Were those lost to follow-up accounted for in the analysis?	Yes	Yes	Yes
10. Did the statistical methods examine changes in outcome measures from before to after the intervention? Were statistical tests done that provided *p* values for the pre-to-post changes?	Yes	Yes	Yes
11. Were outcome measures of interest taken multiple times before the intervention and multiple times after the intervention?	No	No	No
12. If the intervention was conducted at a group level (e.g., a whole hospital, a community, etc.) did the statistical analysis take into account the use of individual-level data to determine effects at the group level?	NA	NA	NA
Overall Quality Rating	**Fair**	**Fair**	**Fair**

**Abbreviations:** CD = Cannot Determine; NR = Not Reported; NA = Not Applicable.

Clinical outcomes were assessed using a broad range of validated psychometric instruments, such as the Beck Depression Inventory (BDI-II), Montgomery–Åsberg Depression Rating Scale (MADRS), Hamilton Anxiety Rating Scale (HAM-A), Positive and Negative Syndrome Scale (PANSS), Scale for the Assessment of Negative Symptoms (SANS), and multiple global and symptom-specific measures. In the first study by Reznik et al., when stimulating the RFTC, no evidence of significant changes was observed over time (F(1,12) = 0.196, p = 0.667), with a very small effect size (ηp^2^ = 0.019), from baseline to 1 month in depressive symptoms when the BDI-II scale was applied. However, the OASIS score improved, indicating a trend towards reduced anxiety at one month of follow-up (F(1,12) = 4.422, p = 0.062), with a large effect size (ηp^2^ = 0.307). The last two scales (PSWQ and RRS) had no reported results. The lack of significance is likely not due to low power, but rather due to the effect itself, which is very small and not statistically significant.

The other scales, even though they were mostly different in each study, showed significant improvement, as shown by the delta scores. In Mahdavi et al., significant reductions in anxiety symptoms were observed at 2 months, with decreases in HAM-A (Δ −12.64) and BAI (Δ −12.88) scores. PGI-I scores indicate that patients have improved substantially (PGI-I = 2.16). Zhaolin et al. showed improvements in negative symptoms (SANS: t = 7.397, p < 0.001) and in the overall psychopathological state (PANSS: F = 4.503, p < 0.05; partial η^2^ = 0.158). Despite that, improvements in cognitive performance were detected on the C-BCT test (F = 4.277, p = 0.050). In the third study by Jooyoung et al., reductions of symptoms were observed at 2 weeks in all the scales applied, which were MADRS (Δ −13.7), QIDS-SR (Δ −10.9), SSI (Δ −10), and K-POMS (Δ −60.8). This indicates rapid improvements in depressive symptoms, suicidal ideation, and overall mood state. The evidence is even stronger since there was a comparative group, and the study's design was double-blinded and randomized. In the final study by Bryan et al., reductions were observed in the overall scales at approximately 3 weeks. These decreases were in general distress (MASQ-GD Δ −5.69), depressive symptoms (PHQ-9 Δ −4.45), anxiety severity (GAD-7 Δ −4.51), and anhedonia (SHAPS).

LIFU was associated with statistically significant reductions in depressive, anxiety, and negative symptom severity, as well as improvements in global functioning and related symptom domains over follow-up periods ranging from weeks to months. However, not all studies were double-blinded. The samples were highly variable, and there was no process in place to address the risk of a placebo effect in patients. The intervention demonstrated a favorable safety profile, with adverse effects being infrequent, mild, and transient, most commonly headache and dizziness, and no serious neurological or psychiatric complications were reported.

Based on the current version of RoB 2, Risk of Bias Tool (Risk of bias tools)

All HIFU studies included in this review were open-label, non-randomized clinical trials with no control group. As such, they were not eligible for formal RoB-2 evaluation and instead were evaluated by the National Institutes of Health (NIH) Quality Assessment Tool for Before–After (Pre–Post) Studies With No Control Group. Overall quality ratings were assigned according to NIH guidance based on methodological strengths and limitations.

## 5 Discussion

This systematic review summarizes the early clinical evidence on transcranial focused ultrasound for psychiatric disorders, including ablative HIFU and nonablative LIFU approaches. Across eight small clinical studies, focused ultrasound interventions were associated with improvements in selected psychiatric rating scales and generally favorable short-term tolerability. However, these findings should be interpreted cautiously because the evidence base remains limited by small sample sizes, diagnostic heterogeneity, variable targets and parameters, short follow-up, and risk of bias. Therefore, current data support focused ultrasound as a promising investigational strategy rather than an established psychiatric treatment.

Studies evaluating LIFU show its utility as an incisionless, reversible neuromodulation technique with minimal adverse effects. Clinical trials applied LIFU to fronto-limbic circuits, including the amygdala and the DLPFC. They had a reduction in depressive, anxiety, and negative schizophrenic symptoms and improved functional and symptom-related domains. However, not all targets were effective. In the Reznik study, during the RFTC stimulation, depressive symptoms showed no meaningful variation with a minimal size effect. Based on these results, one could infer that the RFTC is not an adequate area to be stimulated, since in the other studies, different areas did and obtained better results. Nevertheless, it is important to highlight that the action mechanism of LIFU in the different brain structures is not yet very well understood. Nevertheless, LIFU's favorable tolerability and safety profile make it a potential flexible therapeutic tool for exploratory interventions and for pharmacologically resistant populations.

HIFU has been utilized primarily for movement disorders, such as Parkinson's Disease. In a study conducted by Martinez et al., they found that single-hemisphere ultrasound-guided subthalamotomy improved motor characteristics in selected Parkinson's disease patients with asymmetric signs. However, adverse effects were observed, including speech and gait disturbances, weakness on the treated side, and dyskinesia (Martínez-Fernández et al., 2020). HIFU has proven useful with Essential Tremor (ET). In this randomized controlled trial, which involved 76 patients with medication-refractory ET, transcranial focused ultrasound thalamotomy was applied, reducing hand tremor at 3 months, and the effect persisted during 12-months; however, thalamic lesions appear when performing the procedure, which results in permanent neurologic deficits. These adverse effects were sensory deficits and paresthesias, gait disturbances, cerebellar deficits such as dysmetria, ataxia, and subjective unsteadiness of gait. Despite that, the intensity of adverse events peaked at 1 week after HIFU, matching the maximal size of the lesion with perilesional edema (Elias et al., 2016). Neuropathic pain was also helped by HIFU administration on incisionless central lateral thalamotomies. More than half of the patients (57%) experienced pain relief at 1 year after treatment (Jeanmonod et al., 2012). There exist other emerging indications, such as epilepsy and psychiatric disorders, which have been previously mentioned.

It is important to state that direct comparisons between HIFU and LIFU should be interpreted with caution because both technologies are designed for different therapeutic purposes. HIFU produces permanent thermal lesions through focal ablation and mechanical tissue disruption, permanently altering the pathological circuits (Quadri et al., 2018). This makes HIFU ideal for ablative interventions and well suited for patients with severe, chronic, and treatment-refractory psychiatric disorders. Whereas LIFU aims to reversibly modulate neural circuits without tissue destruction by producing changes in the neuronal membranes, ion channels, and synaptic transmission, causing transient and adjustable effects without structural damage (Cox et al., 2025). Consequently, differences in efficacy or safety cannot be interpreted independently of the clinical indication, stimulation target, and intended therapeutic mechanism.

From a clinical standpoint, these findings suggest that tFUS may occupy a unique position within the possible treatments of psychiatry. HIFU may work as a minimally invasive alternative compared to conventional ablative neurosurgery. However, patients must be carefully selected, with severe, treatment-refractory illness and no recent neurosurgical procedures (Jung et al., 2015b). However, the current evidence suggests that widespread adoption of tFUS should not be used outside specialized centers or research settings. Patient selection, ethical considerations, and multidisciplinary evaluation remain essential.

This review has several limitations. First, the number of eligible clinical studies was small, particularly for HIFU, and most included studies enrolled limited patient samples. Second, substantial heterogeneity was present across diagnoses, neural targets, ultrasound parameters, clinical outcome measures, and follow-up intervals. Third, most HIFU studies were open-label and nonrandomized. They had a high risk of bias, since all had a small sample and no control group. Both the examiner and the participant knew when HIFU was performed, which could cause a placebo effect. Therefore, the foreground study quality may not be ideal. Fourth, no included study directly compared HIFU and LIFU within the same psychiatric population, limiting any direct conclusions regarding comparative efficacy or safety. Finally, the variability of outcome scales and reporting prevented quantitative meta-analysis. These limitations highlight the need for standardized protocols and larger controlled trials before focused ultrasound can be incorporated into routine psychiatric practice.

Future studies should prioritize multicenter, randomized, sham-controlled designs with standardized stimulation or ablation parameters, harmonized outcome measures, and longer follow-up. Trials should also incorporate neuroimaging biomarkers, target-engagement measures, and systematic adverse-event reporting to improve reproducibility and clarify mechanisms of action. Comparative studies against established neuromodulation approaches, including deep brain stimulation, repetitive transcranial magnetic stimulation, vagus nerve stimulation, and electroconvulsive therapy, may help define the appropriate clinical role of focused ultrasound in interventional psychiatry. Until such evidence is available, HIFU and LIFU should be considered investigational approaches for selected psychiatric populations within specialized research settings.

## 6 Conclusions

Preliminary clinical evidence suggests that HIFU and LIFU may be associated with symptom improvement in selected psychiatric populations; however, current evidence remains limited by small samples, heterogeneity, short follow-up, and risk of bias. Larger randomized controlled trials are required to define the efficacy, safety, and optimal clinical role of each modality.

## Authorship statement

All authors made substantial contributions to the conception and design of the study, acquisition of data, analysis and interpretation of data, and drafting or critical revision of the manuscript for important intellectual content. All authors approved the final version of the manuscript submitted for publication and agree to be accountable for all aspects of the work.

## PROSPERO registration record

Available at the following web page:

https://www.crd.york.ac.uk/PROSPERO/view/CRD420261307004.

ID: CRD420261307004.

Date created: 11 February 2026.

Lasted edited: 27 February 2026.

## Source(s) of financial support

This research received no financial aid from any organization, institution, or company.

## Declaration of competing interest

The authors declare that they have no known competing financial interests or personal relationships that could have appeared to influence the work reported in this paper.
