# Supplementary material for: High-intensity focused ultrasound (HIFU) and low-intensity focused ultrasound (LIFU) as neurosurgical approaches in psychiatry: A systematic review of current clinical evidence
Source: Brain Spine. 2026 Sep 7;6:107438. doi: 10.1016/j.bas.2026.107438 (PMC13602696; doi:10.1016/j.bas.2026.107438)
Supplement: Supplementary file 1 [file mmc1.docx]

# Supplementary Table S1. NIH Quality Assessment Tool for Before–After (Pre–Post) Studies With No Control Group

## Jung et al. (2015)

| Criterion | Rating | Justification |
| --- | --- | --- |
| Q1 | **Yes** | The study objective was clearly stated: to evaluate the feasibility, safety, and preliminary efficacy of bilateral MRgFUS capsulotomy in treatment-refractory OCD. |
| Q2 | **Yes** | Inclusion criteria (DSM-IV diagnosis, treatment refractoriness, symptom severity) and exclusion criteria were described. |
| Q3 | **Yes** | Participants represented patients with severe treatment-refractory OCD who would be candidates for this intervention. |
| Q4 | **CD** | The article did not explicitly state whether all eligible patients during the recruitment period were enrolled. |
| Q5 | **No** | Only four participants were included, and no sample size calculation or power analysis was reported. |
| Q6 | **Yes** | The MRgFUS intervention, targeting procedure, sonication parameters, MRI guidance, and postoperative verification were clearly described. |
| Q7 | **Yes** | Outcomes were assessed using validated psychiatric scales (Y-BOCS, HAM-D, HAM-A) administered consistently across participants. |
| Q8 | **No** | The study was open-label, and no blinded outcome assessors were reported. |
| Q9 | **Yes** | All participants completed the planned follow-up, with no evidence of >20% attrition. |
| Q10 | **Yes** | Statistical analyses compared baseline and follow-up scores and reported significance testing. |
| Q11 | **No** | Outcomes were measured once before treatment and multiple times afterward; repeated pre-intervention measurements were not performed. |
| Q12 | **NA** | The intervention was delivered at the individual participant level. |

**Overall Quality:** **Fair**

## Kim et al. (2018)

| **Criterion** | **Rating** | **Justification** |
| --- | --- | --- |
| Q1 | **Yes** | The objective was clearly defined as evaluating the long-term efficacy and safety of bilateral MRgFUS capsulotomy in treatment-refractory OCD. |
| Q2 | **Yes** | Detailed inclusion and exclusion criteria were provided, including DSM-IV diagnosis, treatment history, Y-BOCS threshold, and neuropsychological exclusions. |
| Q3 | **Yes** | Participants were representative of patients with severe treatment-refractory OCD referred to a tertiary center. |
| Q4 | **CD** | Although recruitment procedures were described, the authors did not explicitly state that all eligible patients were enrolled consecutively. |
| Q5 | **No** | Eleven participants were included without a priori sample size or power calculation. |
| Q6 | **Yes** | The surgical procedure, targeting strategy, MRI guidance, sonication parameters, and postoperative imaging were comprehensively described. |
| Q7 | **Yes** | Validated psychiatric scales (Y-BOCS, CGI, HAM-D, HAM-A, GAF) and standardized neuropsychological tests were applied uniformly at prespecified follow-up visits. |
| Q8 | **No** | Outcome assessors were not reported to be blinded; the study was conducted as an open-label trial. |
| Q9 | **Yes** | Follow-up was complete through 24 months, with no substantial loss to follow-up reported. |
| Q10 | **Yes** | Linear mixed-effects models with Bonferroni-adjusted post hoc analyses appropriately evaluated changes from baseline. |
| Q11 | **No** | A single baseline assessment was obtained before intervention, followed by repeated post-treatment evaluations; multiple pre-intervention measurements were not performed. |
| Q12 | **NA** | Individual-level intervention; group-level analysis was not applicable. |

**Overall Quality:** **Fair**

## Davidson et al. (2020)

| **Criterion** | **Rating** | **Justification** |
| --- | --- | --- |
| Q1 | **Yes** | The study objective was clearly stated as evaluating the safety, feasibility, and preliminary efficacy of MRgFUS capsulotomy in OCD and MDD. |
| Q2 | **Yes** | Eligibility criteria for both OCD and MDD cohorts were explicitly described. |
| Q3 | **Yes** | Participants were representative of adults with severe, treatment-refractory OCD or MDD eligible for ablative neurosurgery. |
| Q4 | **CD** | The article described screening and enrollment but did not explicitly report whether all eligible patients were enrolled. |
| Q5 | **No** | Twelve participants were included (6 OCD, 6 MDD), and no formal sample size calculation was reported. |
| Q6 | **Yes** | The intervention protocol, imaging guidance, lesion targeting, sonication parameters, and perioperative procedures were described in detail. |
| Q7 | **Yes** | Validated clinical outcome measures (Y-BOCS, HAMD-17) were administered using standardized protocols throughout follow-up. |
| Q8 | **No** | The study was open-label, and blinded outcome assessment was not reported. |
| Q9 | **Yes** | Follow-up was completed for the enrolled participants with minimal attrition reported. |
| Q10 | **Yes** | Statistical analyses evaluated longitudinal changes from baseline and reported corresponding p-values. |
| Q11 | **No** | Participants underwent one baseline assessment before intervention and repeated follow-up assessments afterward; repeated pre-intervention measurements were not performed. |
| Q12 | **NA** | Individual-level intervention; the group-level criterion did not apply. |

**Overall Quality:** **Fair**

# Summary of Overall Quality

| **Study** | **Overall Quality** | **Main Strengths** | **Main Limitations** |
| --- | --- | --- | --- |
| Jung et al. (2015) | **Fair** | Clear objectives, standardized intervention, validated outcome measures, complete follow-up | Very small sample (n=4), no control group, open-label design, no blinded outcome assessment |
| Kim et al. (2018) | **Fair** | Detailed methodology, long-term (24-month) follow-up, validated assessments, appropriate statistics | Small sample (n=11), open-label, no control group, no blinded assessment |
| Davidson et al. (2020) | **Fair** | Prospective design, standardized intervention, validated outcome measures, appropriate longitudinal analyses | Small sample (n=12), open-label, no control group, no blinded assessment |
